# Classification of the Surrounding Rock Based on Image Processing Analysis and Transfer Learning

**DOI:** 10.3390/jimaging12020089

**Published:** 2026-02-19

**Authors:** Yanyun Fan, Jiaqi Zhu, Hua Luo, Yaxi Shen, Shuanglong Wang, Xiaoning Liu, Dong Li, Chuhan Deng

**Affiliations:** 1 Yunnan Institute of Water and Hydropower Engineering Investigation, Design Co., Ltd., Kunming 650021, China; fanyynsl@163.com (Y.F.); wslynsl@163.com (S.W.); 2 Yunnan Key Laboratory of Water Security, Kunming 650021, China; liuxnynsl@163.com; 3 Yunnan Institute of Water and Hydropower Engineering Investigation Design and Research, Kunming 650021, China; 4 State Key Laboratory of Hydraulic Engineering Simulation and Safety, Tianjin University, Tianjin 300072, China; yaxi@tju.edu.cn; 5 School of Civil Engineering, Tianjin University, Tianjin 300072, China; 6 College of Intelligence and Computing, Tianjin University, Tianjin 300072, China; li_dong@tju.edu.cn

**Keywords:** feature extraction, image processing analysis, surrounding rock classification, box dimension method, transfer learning

## Abstract

Currently, standardized classification methods of surrounding rock are relatively insufficient. The classification of surrounding rock mainly relies on the subjective judgment of technicians, leading to diverse evaluation results. This study focuses on the feature extraction and classification methods of surrounding rock images in a certain tunnel of the Central Yunnan Water Diversion Project by using image processing analysis and transfer learning. Rich surrounding rock images and the water conservancy tunnel data are collected, and then the surrounding rock is classified relatively accurately according to the code and expert guidance. By introducing the fractal theory, the complexity and irregularity of the spatial distribution of weak layers and joints on the surrounding rock surface are revealed effectively. Based on the analysis of changes in fractal dimension characteristic values, a classification method for surrounding rock based on the fractal theory is proposed. Combined with the quantified parameters of surrounding rock images and the strength data collected by rebound meters, a method for correcting the surrounding rock strength based on image analysis is proposed, which can effectively solve the error caused by the uneven distribution of rock masses in the traditional rebound meter strength values. After correction, more accurate strength characteristics can be obtained, which is conducive to the standardized classification of the surrounding rock. After studying the recognition of tunnel surrounding rock images with transfer learning, a model is constructed to achieve rapid classification of tunnel surrounding rock. This research provides support for the standardized classification of tunnel surrounding rock.

## 1. Introduction

The stability of the tunnel surrounding rock is a key factor for ensuring normal construction and personnel safety [1,2]. The risks associated with damage, deformation, and potential geological hazards in the tunnel surrounding rock have become increasingly prominent, making the monitoring and timely evaluation of the surrounding rock particularly important. The evaluation of tunnel surrounding rock serves as a primary basis for designing support structures and surrounding rock parameters [3,4]. In recent years, image processing and recognition technologies have shown considerable potential for tunnel surrounding rock evaluation and classification, owing to their non-contact nature, standardization capability, and effectiveness [5,6,7,8].

Image processing analysis technology can effectively extract key information from the collected surrounding rock surface images and parameterize it through steps such as preprocessing, feature extraction, and analysis [9]. Currently, many scholars focus only on the quantitative analysis of surface parameters using surrounding rock image processing technology, and few apply emerging image theories for further interpretation and analysis. However, the surface features of the tunnel surrounding rock are complex, and quantitative analysis alone is difficult to effectively describe and standardize the evaluation of image features. Utilizing image analysis theories may provide a better approach for in-depth interpretation and analysis of surrounding rock images [10]. Fractal theory is a modern mathematical branch with significant applications in multiple disciplines. It can analyze and evaluate images by revealing self-similarity and fractal dimensions within them. Fractal dimensions can describe the complexity and detail of fractal geometric objects and reflect their filling capacity in space, often exceeding the traditional concept of integer dimensions [11,12]. Huang et al. [13] analyzed the changes in rock freeze–thaw fissures based on fractal theory and evaluated the complexity and distribution of fissure structures using fractal dimensions. As an effective tool for studying nonlinear phenomena and complex structures, fractal theory offers a new perspective and method for effectively describing the complex morphology and texture features of tunnel surrounding rock surfaces [14].

Strength characteristics of the tunnel surrounding rock are critical for its stability [15]. Although traditional methods for obtaining surrounding rock strength can provide detailed mechanical parameters, challenges in practical applications, such as sampling difficulties and non-uniformity, are often encountered. This is particularly evident when the surrounding rock contains interbedded hard rock and weak layers, where weak layers formed by fractures and argillaceous bands are even more difficult to sample [16,17]. In contrast, the rebound hammer method, with its advantages of on-site direct testing and operational simplicity, has been widely used for rapid evaluation of surrounding rock strength in the tunnel. However, when hard rock and weak layers are unevenly distributed, this point-based measurement approach also faces issues of non-uniform measurement [18]. Therefore, due to measurement conditions and the distribution characteristics of the surrounding rock, it is difficult to obtain accurate strength characteristics of the surrounding rock, and the strength values measured on-site are difficult to serve as an important basis for the classification and discrimination of the surrounding rock in practical applications. If the feature parameters extracted from the surrounding rock images through analysis and processing are used to correct the on-site measured strength, more accurate strength characteristics of the surrounding rock can be obtained, thereby improving the effectiveness of the surrounding rock strength measurement.

Deep learning, as an important branch of artificial intelligence, has achieved remarkable progress in the image recognition field due to its powerful feature learning and generalization capabilities [19,20,21]. It can automatically learn image features through multiple layers of abstraction and possesses strong nonlinear mapping capabilities. For tunnel surrounding rock, it can effectively extract complex textures and structural information from surrounding rock images, learning the mapping relationship between surrounding rock images and category labels, thereby improving the classification accuracy and robustness [22,23,24]. However, a key issue with traditional deep learning is that it requires numerous specific domain sample data, especially for evaluating and classifying samples of tunnel surrounding rock structures with high similarity, which requires higher requirements. Moreover, the number of various tunnel surrounding rock photos available for acquisition is relatively limited, especially for numerous and effective samples in the early stage. This is a key issue faced by deep learning of the tunnel surrounding rock. Transfer learning [25], as a training method of deep learning, can effectively solve the classification problem under non-large sample datasets by using pre-trained deep learning models on large-scale datasets as the basis for feature extraction, such as residual neural network (ResNet), etc. It can better and more quickly adapt to the different conditions for tunnel surrounding rock image recognition, greatly simplifying the processing procedure and improving the classification effectiveness [26,27]. Recent studies have further demonstrated the effectiveness of deep learning and transfer learning in tunnel face image analysis and rock strength assessment [28,29,30].

**Figure 1:**
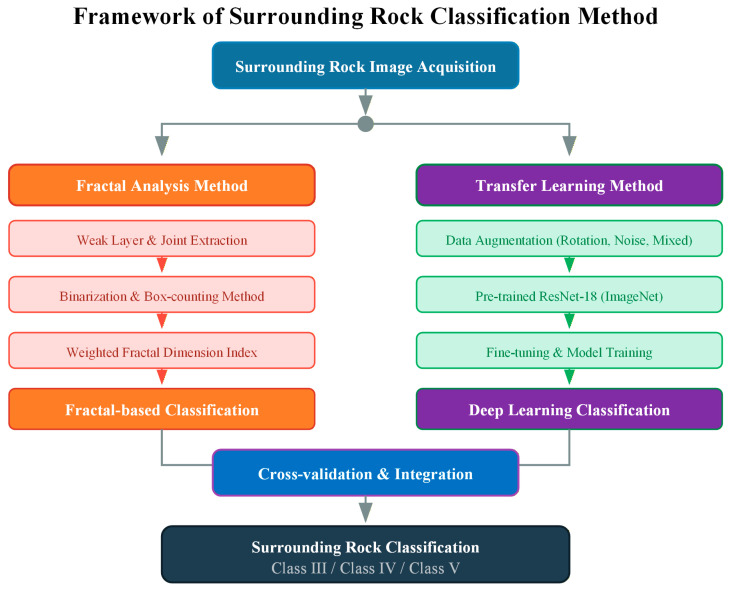
Overall framework of the proposed surrounding rock classification method.

This study aims to research processing analysis and classification techniques for tunnel surrounding rock image, explores characterization and evaluation methodology for surrounding rock based on the fractal theory to extract complex morphological and texture features from rock surfaces. This study examines the correction effects of surrounding rock images on the measurement intensity and the effectiveness of the corrected strength in the classification and investigates the applicability of image recognition based on transfer learning for surrounding rock. The overall framework of the proposed method is illustrated in Figure 1. The framework consists of two parallel analysis approaches that complement each other. The fractal analysis branch extracts weak layers and joints from rock images, calculates fractal dimensions using the box-counting method, and generates a weighted fractal dimension evaluation index for classification. The transfer learning branch employs data augmentation and a pre-trained ResNet-18 model to achieve automated image-based classification. The two approaches provide cross-validation for the surrounding rock classification results, enhancing the reliability and robustness of the final evaluation. When discrepancies occur between the two methods, the more conservative (lower) rock class is adopted to ensure construction safety.

The main contributions of this study are as follows:

(1) A weighted fractal dimension evaluation index is proposed to quantitatively characterize the complexity of weak layers and joints on surrounding rock surfaces, providing a physically interpretable basis for rock classification that differs from purely data-driven approaches.
(2) An image-based strength correction method is developed to address the measurement errors caused by heterogeneous rock mass distribution in traditional rebound hammer testing, which has not been adequately addressed in previous studies.
(3) A transfer learning framework using ResNet-18 is implemented for surrounding rock classification, demonstrating that high accuracy (92.3%) can be achieved with limited training samples through appropriate data augmentation strategies.
(4) The integration of fractal analysis and deep learning provides a dual-validation mechanism, combining the interpretability of traditional methods with the automation capability of modern artificial intelligence techniques.

## 2. Acquisition of Surrounding Rock Images

**Figure 2:**
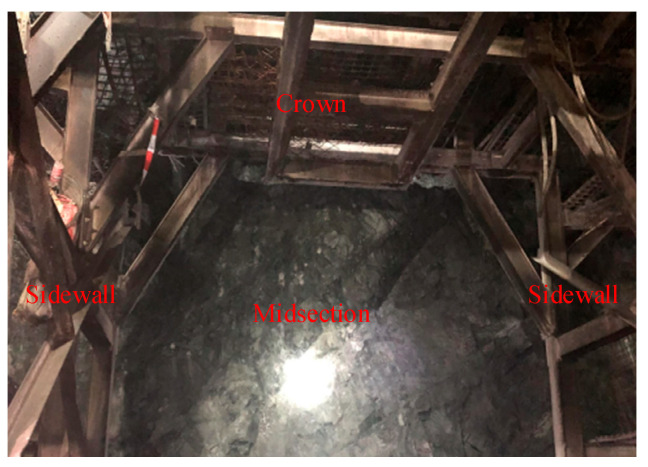
Acquisition position of the standardized tunnel face image.

In accordance with the research objectives, images and strength data were collected from the excavation faces and walls of surrounding rock with different classification grades in a certain tunnel of the Central Yunnan Water Diversion Project. The tunnel geology consists primarily of granite and limestone, with surrounding rock grades mainly categorized as class III, IV, and V, which were rated on-site according to the codes and expert guidance. It should be noted that surrounding rock classification inherently involves a degree of subjectivity, and different experts may assign different grades to the same rock face. To minimize inter-expert variability in this study, the classification was conducted following the standardized criteria specified in the Chinese national code (GB/T 50218-2014: Standard for Engineering Classification of Rock Mass) [31]. Additionally, a consensus-based approach was adopted where experienced engineers discussed and agreed upon the final classification for ambiguous cases. Approximately 1700 faces were finally selected for data acquisition. Standardized images were collected from these faces following a consistent acquisition protocol. The imaging equipment consisted of smartphone cameras (resolution ≥ 12 megapixels) to ensure accessibility and practicality in field conditions. The shooting distance was maintained at 1.5–2.0 m from the rock surface to capture sufficient detail while covering an adequate area. Illumination was provided by the tunnel’s permanent LED lighting system to ensure consistent lighting conditions across all images. The camera was positioned perpendicular to the rock surface to minimize perspective distortion. Collection areas covered the midsection, crown, and sidewall of each excavation face, as illustrated in Figure 2. Each image was manually reviewed to exclude samples with motion blur, severe shadows, or water interference.

**Figure 3:**
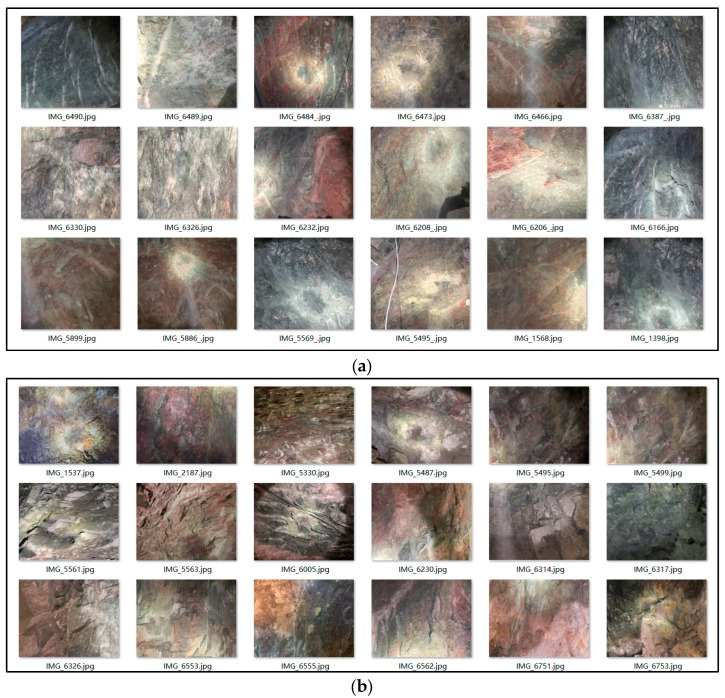

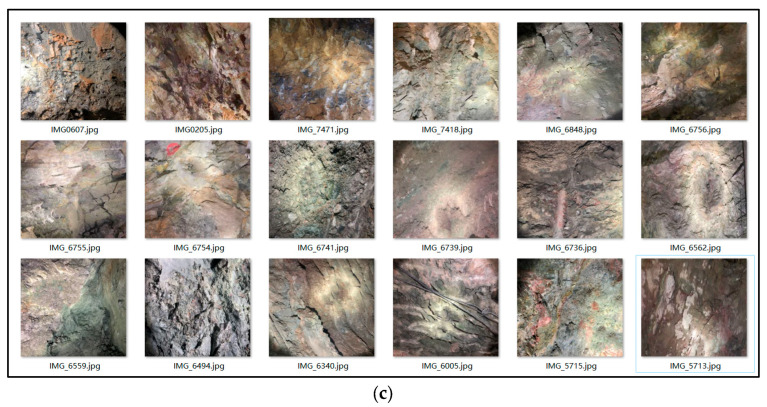
Representative images of various classes of surrounding rock. (**a**) Class III; (**b**) class IV; (**c**) class V.

Approximately 3800 images of the surrounding rock were collected, and parts belonging to different rock classes are shown in Figure 3. class III surrounding rock shows relatively undeveloped joints, an intact internal structure, and sparse fracture distribution. In contrast, class IV surrounding rock displays a more complex structure with well-developed joints, occasionally containing minor weak surfaces. Meanwhile, class V surrounding rock is characterized by fully developed and widely distributed joints, and a clearly visible weak surface in certain areas. Collectively, these features provide a crucial basis for the classification and stability evaluation of the surrounding rock.

The degree of joint development and the distribution of weak surfaces vary significantly across different classes of surrounding rock. These characteristics serve as a critical basis for surrounding rock classification and stability evaluation. However, quantifying these characteristics remains a key focus of current research. Establishing a quantitative characterization system and evaluation method is of great significance. It not only deepens the understanding of surrounding rock characteristics but also provides practical guidance for engineering and is the key to improving the evaluation standards for surrounding rock.

## 3. Discrimination and Analysis of Surrounding Rock Joints Based on Fractal Theory

### 3.1. Fractal Features of Various Class Surrounding Rock Joints

Fractal theory provides a powerful tool for image analysis, effectively revealing the complexity of features on excavated rock surfaces, mud bands, and joints in the surrounding rock excavation. It can also reflect the structure, development and distribution of joints on the surrounding rock surface through the joint conditions and evaluate the structural characteristics of the surrounding rock surface image. The fractal theory describes complex geometric shapes using non-integer dimensions. The fractal box dimension method, as one of the fractal dimensions, is an important tool for quantifying the complexity of fractal structures. The box-counting method calculates by counting the number of the smallest square grids covering the object. If the number of boxes required increases at a certain fixed rate as the grid size decreases, then the logarithm of this rate is the box dimension of the fractal. The box dimension can be calculated by the following formula:

(1) $$D=\underset{\varepsilon \to 0}{\lim}\frac{\log(N(\varepsilon ))}{\log(1/\varepsilon )}$$

where *D* is the fractal dimension, N(ε) is the box number, and *ε* is the box size.

**Figure 4:**
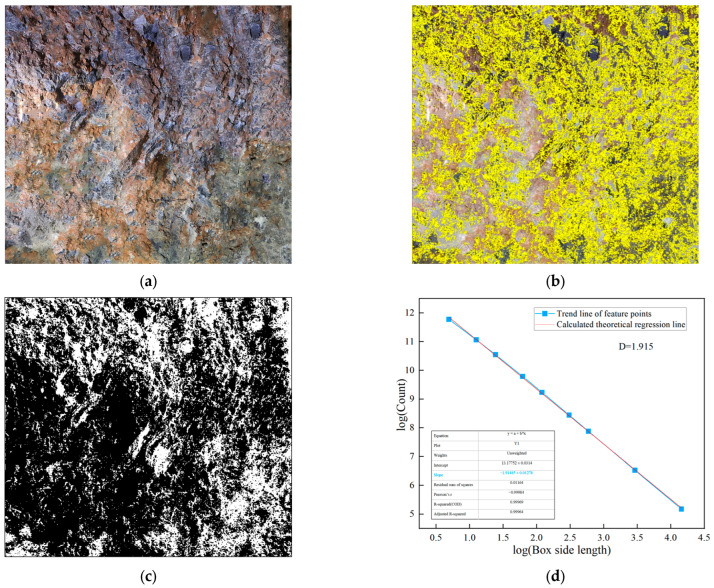
The image processing analysis of class V surrounding rock. (**a**) Class V surrounding rock; (**b**) weak surface extraction; (**c**) binarization processing; (**d**) fractal dimension analysis.

**Figure 5:**
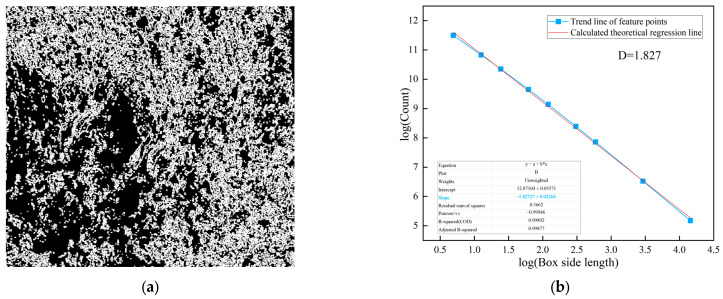
Joint diagram analysis of class V surrounding rock. (**a**) Extraction of joints in class V surrounding rock; (**b**) fractal dimension analysis.

In practice, the fractal dimension is computed through linear regression analysis on a log-log plot. The image is covered with square boxes of varying sizes ε, and the number of boxes *N*(*ε*) required to cover the target features (weak layers or joints) is counted for each box size. By plotting log(1/*ε*) on the *x*-axis against log(*N*(*ε*)) on the *y*-axis, a linear relationship is obtained. The slope of the fitted regression line represents the fractal dimension *D*, as shown in Figure 4d and Figure 5b, and subsequent fractal analysis figures. A higher slope indicates greater structural complexity and irregularity of the analyzed features. The coefficient of determination (*R*^2^) of the linear regression, typically exceeding 0.99 in this study, confirms the validity of the fractal characterization for the surrounding rock images.

Class V surrounding rock exhibits various complex matrix planes and structural features. Weak layers formed by mud bands, joints, etc., significantly affect the overall stability of the surrounding rock. The complexity of these weak layers can be effectively characterized using fractal theory. The weak layer extraction was performed using the following image processing procedure. First, the original RGB image was converted to grayscale. Then, Gaussian filtering (kernel size 5 × 5) was applied to reduce the noise while preserving edge features. The weak layers were segmented from the intact hard rock using Otsu’s automatic thresholding method, which determines the optimal threshold by maximizing the between-class variance of pixel intensities. This technique is well-established in image processing and is particularly effective for images with bimodal intensity distributions, such as surrounding rock images containing distinct weak layers and hard rock regions. Finally, morphological operations (opening and closing with a 3 × 3 kernel) were applied to remove small noise artifacts and fill minor gaps. The resulting binary image clearly delineates the weak layer regions, as shown in Figure 3b,c. Within the weak layer regions in the images, the box dimension method based on fractal theory is used to investigate their complexity and structural properties. The fractal analysis results for class V surrounding rock are shown in Figure 4.

To ensure consistency and reproducibility of the fractal analysis, all images were processed using standardized parameters. The original images were resized to a uniform resolution of 512 × 512 pixels before analysis. The Otsu’s thresholding method was employed to automatically determine the optimal binarization threshold for each image, eliminating subjective bias in threshold selection. For the box-counting analysis, box sizes ranging from 2 to 64 pixels (2, 4, 8, 16, 32, 64) were used, which is a widely accepted range in fractal dimension calculations. The high coefficients of determination (*R*^2^ > 0.99) obtained in the log-log regression analysis confirm the reliability of the fractal dimension calculations under these standardized conditions.

The lines on the surrounding rock surface are an important source of information reflecting joint composition and development complexity. Using image processing techniques, the lines formed by the development of joints on the surrounding rock surface are extracted. The complexity and irregularity characteristics of the joints on the surrounding rock surface are analyzed using the box dimension method, as shown in Figure 5.

**Figure 6:**
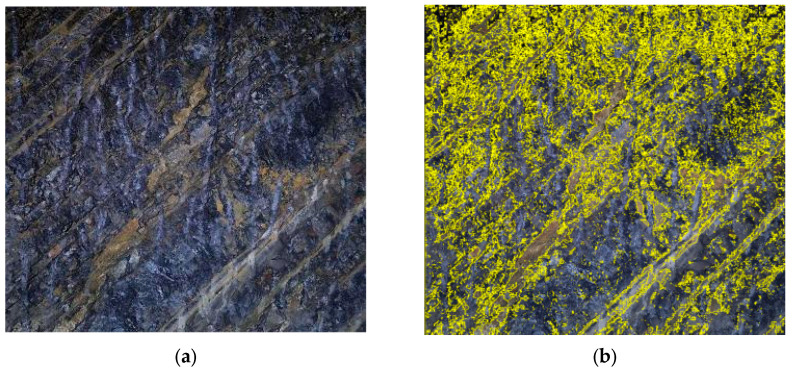

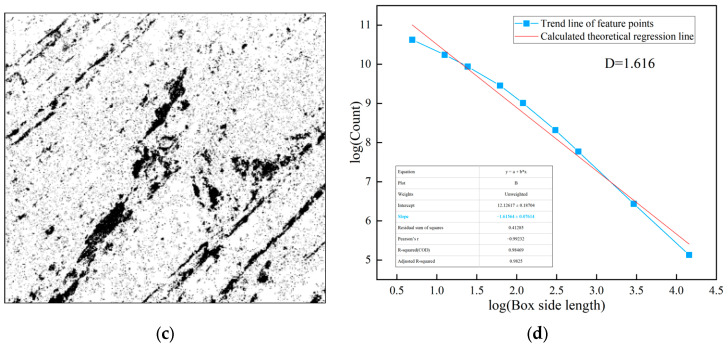
Image fractal processing in class IV surrounding rock. (**a**) Class IV surrounding rock; (**b**) weak surface extraction; (**c**) binarization processing; (**d**) fractal dimension analysis.

**Figure 7:**
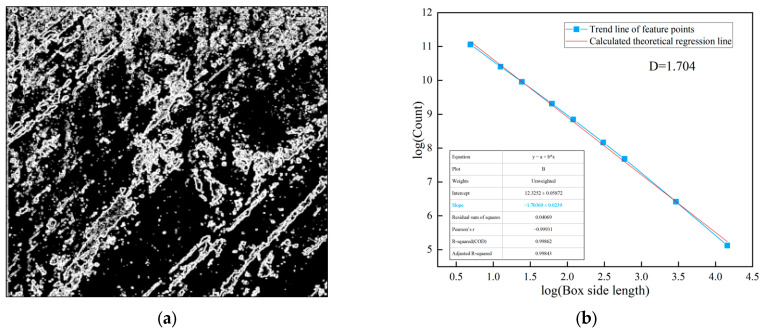
Image fractal processing analysis in class IV surrounding rock. (**a**) Extraction of joints in class IV surrounding rock; (**b**) fractal dimension analysis.

As shown in Figure 4 and Figure 5, the fractal dimension of the weak layer in this class V surrounding rock is 1.915, while that of the joint surface is 1.827. Both values are relatively high, indicating a significant degree of structural complexity within the class V surrounding rock. Further fractal analysis is performed on the weak layer patterns and surface joints of class IV and class III surrounding rocks. This aims to systematically reveal the differences in matrix distribution and joint structural complexity among different surrounding rock classes, thereby providing a more detailed and accurate basis for image-standardization evaluation in surrounding rock classification. The processing and analysis results for class IV surrounding rocks are shown in Figure 6 and Figure 7.

From Figure 6 and Figure 7, it can be seen that the fractal dimension of the weak layer in this class IV surrounding rock is 1.616, and the fractal dimension of the joint is 1.704. Compared with the previous class V surrounding rock, there is a significant downward trend. When adding the fractal dimension of the weak layer in the surrounding rock image and the fractal dimension of joints to obtain the overall fractal dimension characteristics, it is found that the value of this class IV surrounding rock is 3.32, which is lower than the 3.742 of the previous class V surrounding rock. This indicates that the overall complexity and irregularity of the structural form of the weak layer and joints in this class IV surrounding rock have decreased.

**Figure 8:**
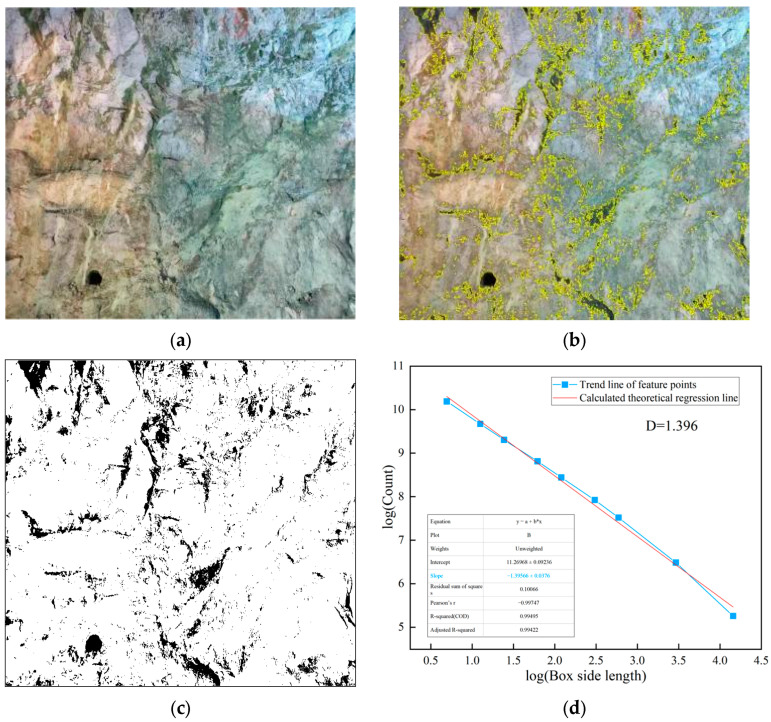
Image Fractal Processing in class III Surrounding Rock a. (**a**) Class III surrounding rock a; (**b**) weak layer extraction; (**c**) binarization processing; (**d**) fractal dimension analysis.

**Figure 9:**
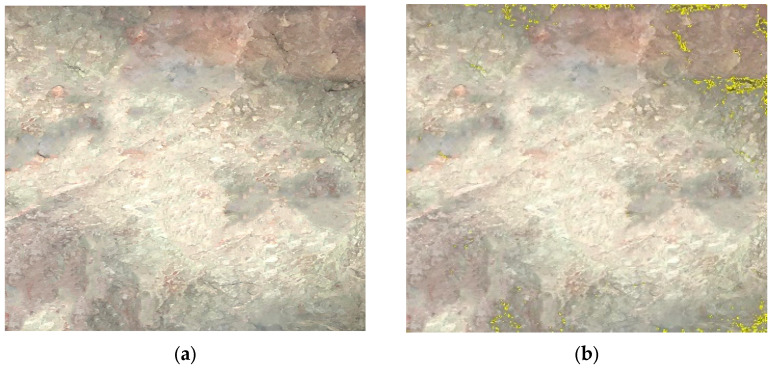

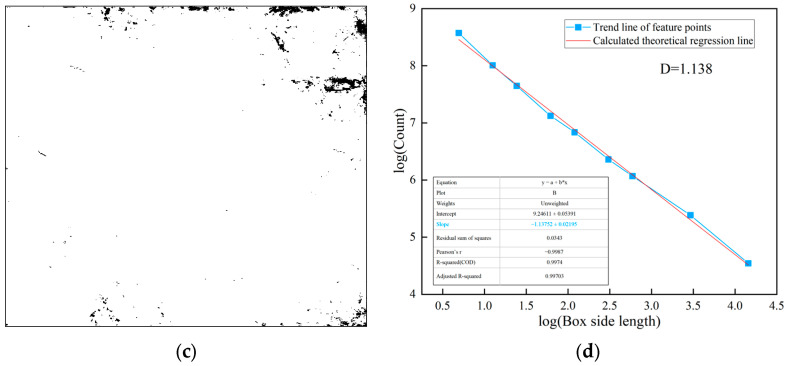
Fracture Processing of class III surrounding rock b. (**a**) Class III surrounding rock b; (**b**) weak layer extraction; (**c**) binarization processing; (**d**) fractal dimension analysis.

**Figure 10:**
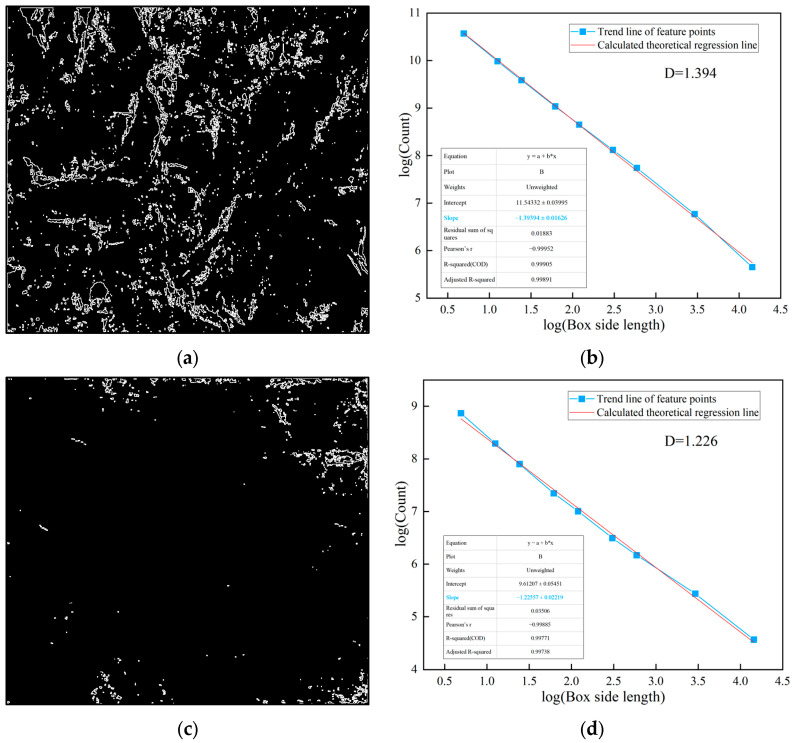
Fractal Processing of class III surrounding rock a, b. (**a**) Joint extraction of class III surrounding rock a; (**b**) fractal analysis of joints in class III surrounding rock a; (**c**) joint extraction of class iii surrounding rock b; (**d**) fractal analysis of joints in class III surrounding rock b.

The processing and analysis procedure of the weak layer graphics and joint line diagrams of the two class III surrounding rocks are shown in Figure 8, Figure 9 and Figure 10. As can be seen from the figures, the fractal dimension of the weak layer of class III surrounding rock a is 1.396, and the fractal dimension of the joint diagram is 1.394; the fractal dimension of the weak layer of class III surrounding rock b is 1.138, and the fractal dimension of the joint line diagram is 1.226. The fractal dimensions of the weak layer and joint line diagrams of class III surrounding rock a are both greater than those of class III surrounding rock b, indicating that the integrity degree of class III surrounding rock a is worse than that of class III surrounding rock b. However, the fractal dimensions of both class III surrounding rocks are significantly smaller than those of class IV and V surrounding rocks.

### 3.2. Discrimination and Evaluation of Fractal Feature in Surrounding Rock

**Figure 11:**
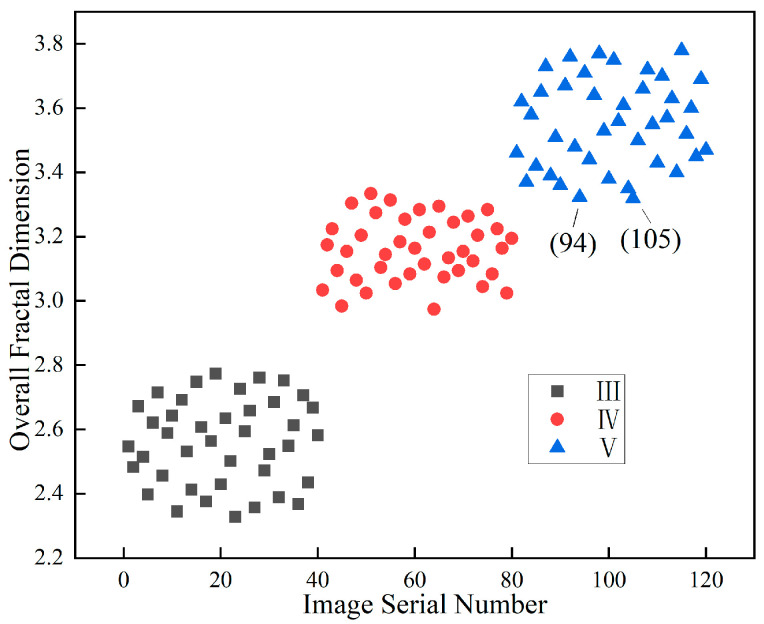
Distribution range of the overall fractal dimension of various class surrounding rocks.

From the previous content, it can be seen that there is a significant correlation between the surrounding rock classification and the fractal dimensions of the image’s weak layer and joint. Both the fractal dimension of the image’s weak layer, the fractal dimension of the joints, and their sum as a whole fractal dimension all follow the pattern that the poorer the surrounding rock quality, the larger the fractal dimension. The overall fractal dimension is more comprehensive and can better reflect the structural characteristics of the image. This study processed and analyzed more surrounding rock images, obtaining the overall fractal dimension distribution of 120 photos of the surrounding rock. The specific results are shown in Figure 11.

As shown in the figure, distinct differences are observed in the overall fractal dimension distribution ranges of classes III, IV, and V surrounding rock. The overall fractal dimension range of class III surrounding rock mainly falls within the interval of 2.321 to 2.786, while that of class IV ranges mainly from 2.974 to 3.344, and that of class V ranges from 3.322 to 3.884. The overall change in trend is that the worse the surrounding rock, the higher the overall fractal dimension. Meanwhile, a clear boundary exists in the distribution range of the overall fractal dimensions of classes III and IV, that is, the maximum value of class III is 2.786 and that of class IV is 2.974. Both classes IV and V also have differences in the distribution range, but there are some values with smaller fractal dimensions in class V that are less than the maximum value of class IV surrounding rock, and the range boundary is not clear.

### 3.3. Research and Correction Analysis on Fractal Feature Points

**Figure 12:**
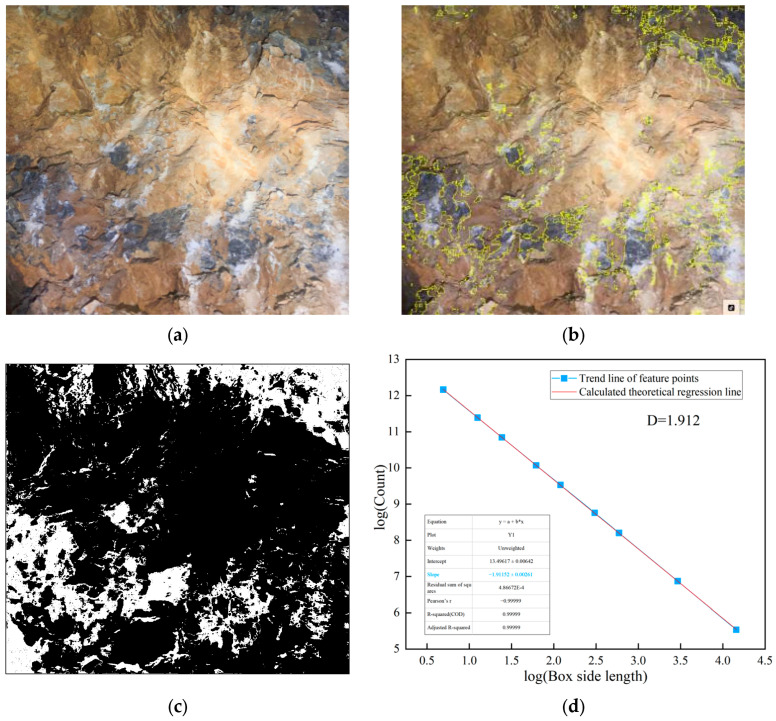
Processing analysis of the 94th surrounding rock image (class V). (**a**) Class V surrounding rock; (**b**) weak surface extraction; (**c**) binarization processing; (**d**) fractal dimension analysis.

**Figure 13:**
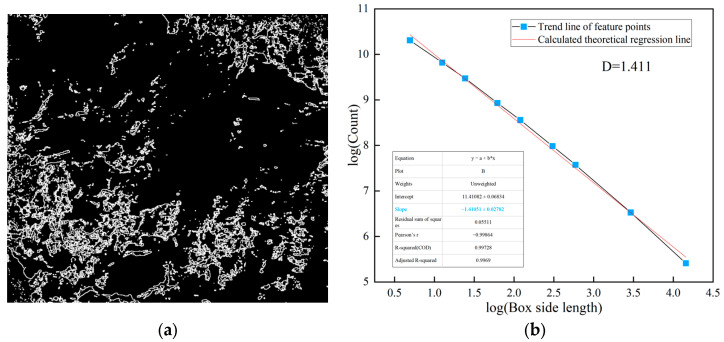
Joint analysis of the 94th surrounding rock image (class V). (**a**) Extraction of joints in class V surrounding rock (**b**) fractal dimension analysis.

The analysis was conducted on the 94th and 105th images, which had the lowest overall fractal dimension values among class V samples. It was found that the common feature of these two surrounding rock images was that the distribution range of weak argillaceous strips was relatively wide. The fractal dimensions of these weak areas are both relatively high, at 1.912 and 1.909. However, no significant joints were extracted from the surface of the weak argillaceous strips, which led to the surface line fractal dimensions being lower than class IV, only 1.411 and 1.416. Therefore, the sum of these two images was only 3.323 and 3.325. The processing and analysis of image 94 are shown in Figure 12 and Figure 13.

**Figure 14:**
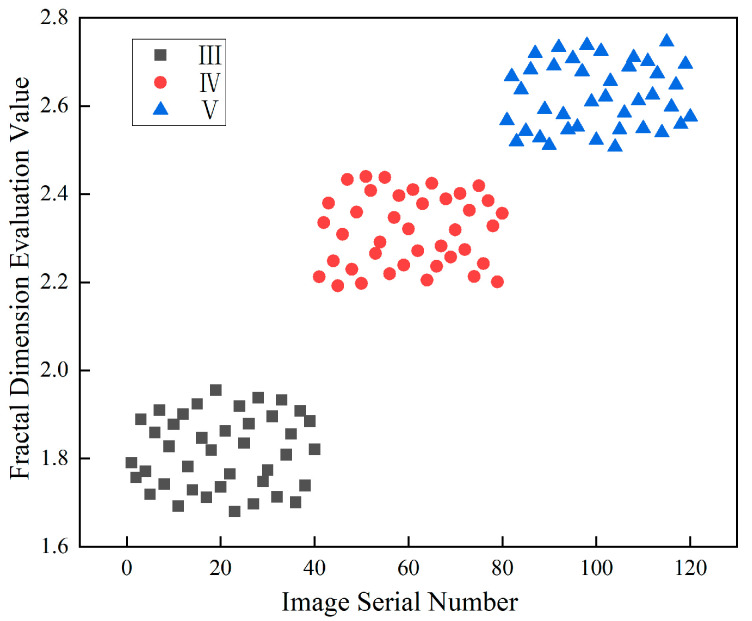
Distribution range of overall fractal dimension evaluation values for various surrounding rocks.

To address the Special Issue of the fractal dimension of class V surrounding rock with a high distribution of mud-rich bands, considering the weak layers and joints at the site, the influence of the weak surfaces on the stability of the surrounding rock is greater. The weights of the fractal dimension for evaluating the fractal dimension of the weak layer shape and the fractal dimension of the joint are adjusted. A fractal dimension evaluation index is proposed to represent this. The fractal dimension evaluation index is set as the sum of the fractal dimension of the weak layer and 0.45 times the fractal dimension of the joint. The weighting coefficient of 0.45 was determined through the following data-driven approach: (1) Engineering rationale: Based on field observations, weak layers (mud bands, fractured zones) have a more significant impact on surrounding rock stability than joints, as they represent continuous zones of structural weakness. (2) Regression analysis: Multiple linear regression was performed on 120 rock samples using the surrounding rock class as the dependent variable. The analysis indicated that the contribution of weak layer fractal dimension to rock classification is approximately 2.2 times that of the joint fractal dimension. To simplify the evaluation index while preserving this relative weighting, the coefficient for joints was set to 0.45 (≈1/2.2), with the weak layer coefficient normalized to 1.0. (3) Validation: The effectiveness of this coefficient was verified by the clear separation of class boundaries shown in Figure 14.

Figure 14 presents the weighted fractal dimension evaluation indices calculated for all 120 surrounding rock images (class III: n = 40, class IV: n = 42, class V: n = 38). As shown in the figure, the weighted fractal dimension evaluation indices of different surrounding rock exhibit notable differences and distinct distribution ranges. The special point of the value for class V surrounding rock returned to the normal range. Following the weighting adjustment, the previously anomalous value observed for class V surrounding rock has returned to the normal range. This anomaly originally arose due to a significant discrepancy between the fractal dimension of its weak-layer pattern and that of its joints, whereas for normal data points, the two dimensions generally vary consistently—both tend to increase as rock quality worsens.

After the weighting process, values at normal points typically decrease overall. In contrast, for special points characterized by a high weak layer fractal dimension but a low joint line fractal dimension, the composite index increases relative to other points. This results in clearly separated distribution ranges for each rock class.

Specifically, the fractal dimension evaluation index ranges are 1.684–1.955 for class III surrounding rock, 2.192–2.443 for class IV, and 2.507–2.745 for class V. These ranges show well-defined classification boundaries, enabling effective differentiation of surrounding rock conditions. This approach provides a reliable method for the standardized classification and evaluation of surrounding rock surface images.

## 4. Strength Correction and Classification Method Based on Image Quantitative Analysis

**Figure 15:**
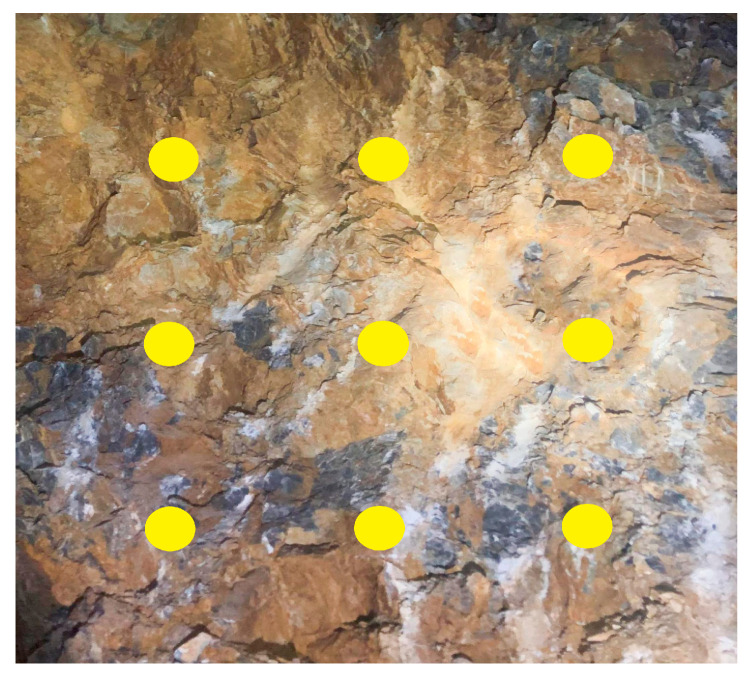
Schematic diagram of rebound hammer measuring points.

The rebound hammer method is widely used for the rapid evaluation of surrounding rock strength due to its straightforward on-site application and operation simplicity. However, when hard rock and weak layers are unevenly distributed within the surrounding rock, the conventional point-based measurement approach tends to yield uneven results, making it difficult to accurately characterize the surrounding rock strength. In class IV and class V surrounding rock, the presence of weak layers such as fractured zones or argillaceous bands is relatively common. For such surrounding rock, the rebound hammer can still reflect the strength to some extent by applying an averaging method for both measurement points and recorded values, as illustrated in Figure 15.

Each measurement point yields an individual strength value. After discarding excessively high and low outliers, the average of the valid readings is taken as the final result, calculated using Equation (2).

(2) $$R_{m}=\frac{1}{n}\sum_{i=1}^{n}R_{i}$$

where $R_{m}$ is the average rebound strength value (MPa), $R_{i}$ is the rebound value at the *i*-th measurement point (MPa), and n is the total number of valid measurement points.

**Figure 16:**
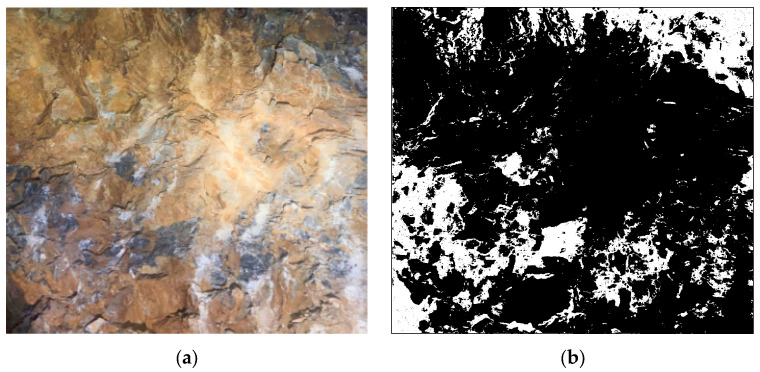
Quantification of weak layer area proportion. (**a**) Class V surrounding rock b; (**b**) area proportion 74.658%.

However, when hard rock and weak layers are unevenly distributed within the test region, the rebound hammer measurements can also be affected, meaning the obtained data may not directly support surrounding rock classification. To address this issue, this study investigates a strength classification method for surrounding rock by integrating quantitative image analysis with rebound hammer testing. Through image recognition and quantitative analysis techniques, the distribution zones of hard rock and weak layers are identified. Combined with multiple-point strength data from the rebound hammer, regionally weighted correction is applied to the original measurements to reduce errors caused by lithological heterogeneity. The weak layers are delineated and their proportional area within the rock image is calculated, as shown in Figure 16, where the black regions represent weak layers accounting for 74.658% of the total area.

**Figure 17:**
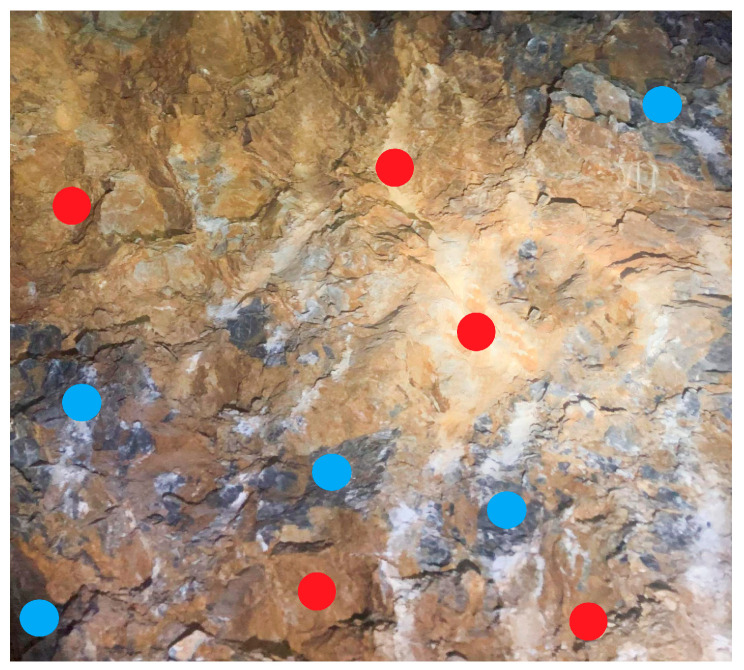
Schematic diagram of rebound hammer testing on heterogeneous surrounding rock.

Strength correction is performed based on the area proportions of hard rock and weak layers. The proposed method is based on the following assumptions: (1) Linear weighting assumption: The overall strength of heterogeneous surrounding rock can be approximated as a linear combination of the strength values of hard rock and weak layer regions, weighted by their respective area proportions. This assumption is reasonable for engineering applications where a representative bulk strength is required. (2) Independence of measurement points: Each rebound hammer measurement point is assumed to provide an independent strength estimate for its local region (hard rock or weak layer). The spatial correlation between adjacent points is considered negligible given the typical measurement spacing. (3) Area-strength proportionality: The surface area proportion of weak layers visible in the 2D image is assumed to be representative of their volumetric contribution to the overall rock mass strength. The specific procedure involves obtaining the rebound strength values of hard rock and weak layers separately, as shown in Figure 17. In the figure, red points represent measurement points within the weak layers, while blue points denote those in the hard rock. After extraction, the strength values obtained from the weak layer and hard rock areas are averaged separately. The corrected strength of the surrounding rock is then calculated as the weighted sum of the average strength values of the weak and hard rock areas, weighted by their respective area proportions. The correction formula is as follows:

(3) $$R_{c}=A_{y}\times \frac{1}{n_{y}}\sum_{i=1}^{n_{y}}R_{yi}+A_{t}\times \frac{1}{n_{t}}\sum_{i=1}^{n_{t}}R_{ti}$$

where $R_{c}$ is the corrected rebound strength value (MPa), $A_{y}$ is the area proportion of hard rock in the measurement area (dimensionless, ranging from 0 to 1), $n_{y}$ is the number of measurement points in the hard rock zones, $R_{yi}$ is the rebound value at the *i*-th point in hard rock (MPa), $A_{t}$ is the area proportion of the weak layer in the measurement area (dimensionless, ranging from 0 to 1), $n_{t}$ is the number of measurement points in the weak layer zones, and $R_{ti}$ is the rebound value at the *i*-th point in the weak layer (MPa). Note that $A_{y}$ + $A_{t}$ = 1.

**Figure 18:**
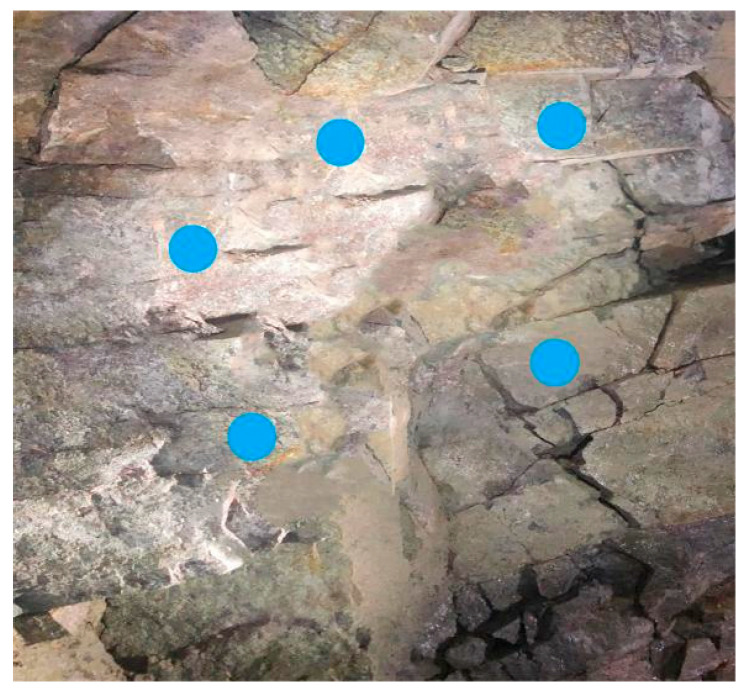
Schematic diagram of rebound hammer testing in hard rock zones.

When the weak layer contains extremely soft argillaceous filling or consists of fractured cavities, as illustrated in Figure 18, the rebound hammer cannot obtain reliable strength measurements from these zones. Nevertheless, the area proportions of the weak zones (including cavities) and the hard rock regions can still be quantified. Under such surrounding rock conditions, the corrected strength is calculated as the product of the average strength value of the hard rock zone and its corresponding area proportion, using the following formula:

(4) $$R_{c}=A_{y}\times \frac{1}{n_{y}}\sum_{i=1}^{n_{y}}R_{yi}$$

where $R_{c}$ is the corrected rebound strength value (MPa), $A_{y}$ is the area proportion of hard rock (dimensionless, ranging from 0 to 1), $n_{y}$ is the number of measurement points in the hard rock zones, and $R_{yi}$ is the rebound value at the *i*-th point in hard rock (MPa).

**Figure 19:**
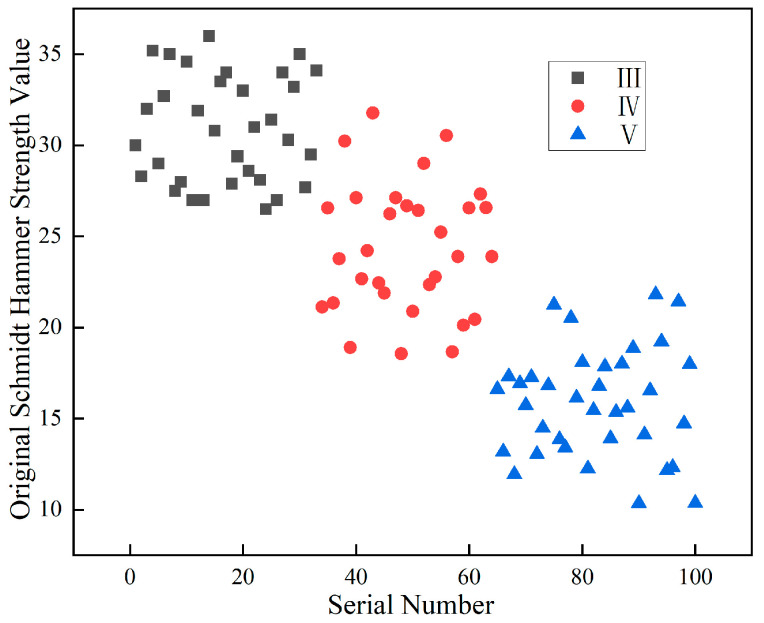
Original strength values from rebound hammer testing.

The proposed correction method was applied to analyze the rebound hammer strength data collected from the surfaces of various classes of surrounding rock. A total of 100 sets of rock surface images and corresponding strength data were utilized for validating the strength correction method. It should be noted that these 100 sets are distinct from the 3800 images used for the deep learning classification task (Section 5). The 100 sets specifically refer to cases where both image data and multi-point rebound hammer measurements were simultaneously collected from the same rock face, enabling the validation of the proposed strength correction method. Each set contains one rock surface image paired with multiple rebound hammer readings. The sample sizes for each rock class are class III (n = 32), class IV (n = 38), and class V (n = 30), providing sufficient samples to demonstrate the distribution characteristics and the effectiveness of the correction method across all rock classes. The uncorrected average point-wise rebound strength values of these samples, categorized by rock class, are shown in Figure 19. The measured strength ranges are 26.4–36.1 for class III, 18.5–31.7 for class IV, and 10.3–21.8 for class V. Although the data generally exhibit a decreasing trend from class III to class V, significant overlapping intervals are observed between adjacent surrounding rock classes. This substantial overlap indicates that the uncorrected rebound hammer strength values alone cannot serve as a reliable basis for clear classification.

**Figure 20:**
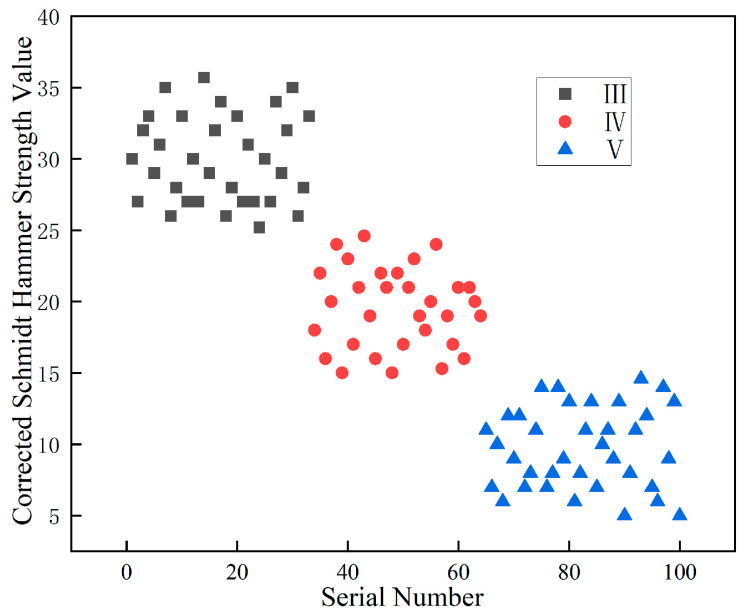
Corrected Schmidt hammer strength values.

Based on the aforementioned correction method, the original rebound hammer strength values were processed and adjusted using image quantification techniques. The results after correction are shown in Figure 20. As can be seen from the figure, the corrected strength ranges are 25.2–35.7 for class III, 14.9–24.6 for class IV, and 5.1–14.7 for class V. The corrected values exhibit a more pronounced decreasing trend from class III to class V, with well-defined distribution ranges and clear boundaries between rock classes. This demonstrates that the corrected strength values better reflect the strength characteristics of different rock classes and provide a more effective basis for classification.

To quantitatively assess the improvement in class separation, the overlap between adjacent rock classes was calculated. Before correction, the overlap between class III and class IV was 5.3 MPa (ranging from 26.4 to 31.7 MPa), and the overlap between class IV and class V was 3.3 MPa (ranging from 18.5 to 21.8 MPa), resulting in a total overlap of 8.6 MPa. After correction, the overlap between class III and class IV was eliminated (class III minimum 25.2 MPa > class IV maximum 24.6 MPa), and the overlap between class IV and class V was reduced to only 0.2 MPa. The total overlap reduction reached 97.7%, demonstrating the significant effectiveness of the proposed correction method in improving class separability. The effectiveness of the proposed correction method was further validated by comparing it with the traditional averaging method. The traditional method calculates the mean strength value across all measurement points without considering the spatial distribution of hard rock and weak layers, which is equivalent to the uncorrected values shown in Figure 18. In contrast, the proposed area-weighted correction method accounts for the heterogeneous distribution of rock materials. The comparison demonstrates that the proposed method significantly outperforms the traditional averaging approach, achieving complete separation between classes III and IV and reducing total class overlap by 97.7%.

The method of correcting rebound hammer strength values through image quantitative analysis enables comprehensive consideration of the distinct characteristics of hard rock and weak layer, yielding more effective assessment data for surrounding rock strength. This corrected strength value more accurately reflects the actual strength state of the surrounding rock, making it better suited for application in surrounding rock classification and tunnel support design.

## 5. Surrounding Rock Classification and Recognition Based on Transfer Learning

Deep learning has distinct advantages in image-based classification tasks, as it can automatically learn discriminative features from images and process complex textures and structural information, thereby enhancing classification accuracy and robustness. However, its practical applicability in tunnel engineering is often constrained by the limited availability of annotated surrounding rock images, which restricts the performance of traditional deep learning models that typically require a large-scale dataset. To overcome this limitation, this study adopts a transfer learning approach. By leveraging a pre-trained model (ResNet-18) and applying transfer learning techniques, the method reduces both the demand for large datasets and the computational burden. Through fine-tuning parameter, the model achieves efficient and accurate identification of surrounding rock classes, demonstrating strong adaptability for engineering applications. This research focuses on developing an intelligent recognition method for surrounding rock classification based on deep ResNet and transfer learning.

### 5.1. Dataset Construction

**Figure 21:**
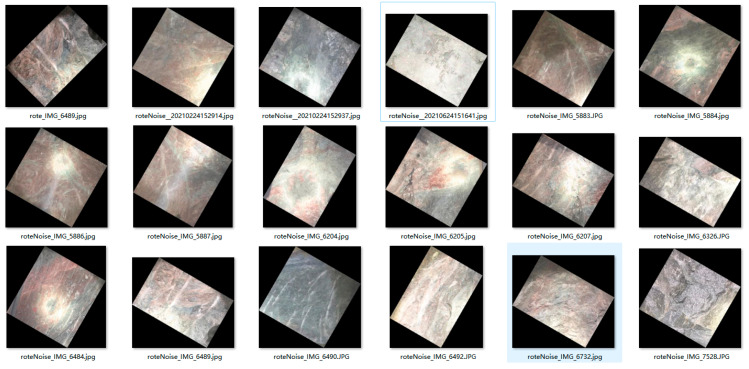
Partial image after data augmentation.

To mitigate the issue of insufficient sample data, data augmentation techniques were employed. The core principle of data augmentation is to generate additional training images through various transformation operations without altering their original categories. This study utilizes rotation, noise addition, and mixed augmentation methods for data augmentation, as shown in Figure 21. After data augmentation, the total number of training samples increased from over 3800 to 11,280, thereby significantly enhancing the data foundation for model training.

The class distribution of the dataset after augmentation is as follows: class III (3756 images), class IV (4128 images), and class V (3396 images). To address the slight class imbalance, stratified sampling was employed during data splitting to ensure that each class maintains the same proportion in both training and testing sets.

### 5.2. Model Construction and Training Process

This study adopts the ResNet-18 model based on transfer learning to rapidly and effectively identify and classify images of surrounding rock. This study utilizes the pre-training results of ResNet-18 in the large database (ImageNet), and the pre-trained feature results obtained in the large database are applied to the identification of tunnel surrounding rock images through transfer learning. This will significantly reduce the training time and the overfitting risk and enhance the model’s generalization ability. It will be better suited for the small sample complex data features of the surrounding rock in the early stages of various engineering projects.

**Figure 22:**
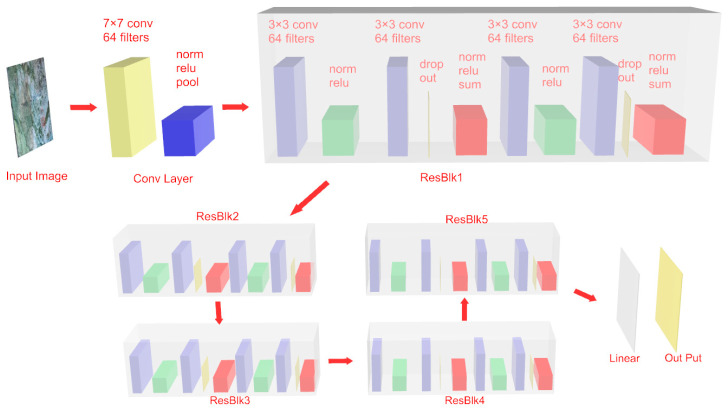
Transfer learning network structure.

The structure of ResNet-18 is shown in Figure 22. The 7 × 7 convolution layer and the initial convolution layer with 64 filters are used to conduct preliminary feature analysis and extraction on the collected rock surrounding images. The model incorporates the residual block (ResBlock) structure, with each residual block having a double 3 × 3 convolution and a residual connection. The convolution layer is followed by normalization and ReLU activation functions to enhance the nonlinear expression. The residual connection alleviates the gradient disappearance problem in the training of deep networks through identity mapping, improving the model performance. After being processed by multiple residual blocks in series, the features finally reach the linear output layer to generate the final prediction result.

To improve the model’s accuracy, the dataset is first partitioned into a training set and a testing set in a 7:3 ratio. The Adam optimizer is selected to adjust the model’s hyperparameter, with an initial learning rate of 0.001, a batch size of 32, and the cross-entropy loss function. Training is performed over 50 epochs. The dataset splitting employed stratified sampling to maintain consistent class distributions in both training and testing sets. To control overfitting, multiple strategies were implemented: (1) Data augmentation (rotation, noise, mixed) to increase sample diversity; (2) Transfer learning with pre-trained weights to reduce the number of parameters requiring training from scratch; (3) Early stopping with patience of 10 epochs to prevent over-training; (4) Dropout layer (rate = 0.5) added before the final fully connected layer.

The model was implemented using PyTorch 1.12 framework with Python 3.8. Training was conducted on a workstation equipped with an NVIDIA GeForce RTX 3080 GPU (10 GB memory). For the transfer learning strategy, the ResNet-18 model pre-trained on ImageNet was used as the backbone. The convolutional layers (conv1 through layer4) were initialized with pre-trained weights and set to trainable mode with a reduced learning rate (0.0001) to preserve learned features while allowing fine-tuning. The fully connected layer was replaced with a new layer matching the number of rock classes (3 classes) and trained with the standard learning rate (0.001). This differential learning rate strategy enables the model to adapt to the specific features of surrounding rock images while retaining the general feature extraction capabilities learned from ImageNet.

### 5.3. Result and Analysis

**Figure 23:**
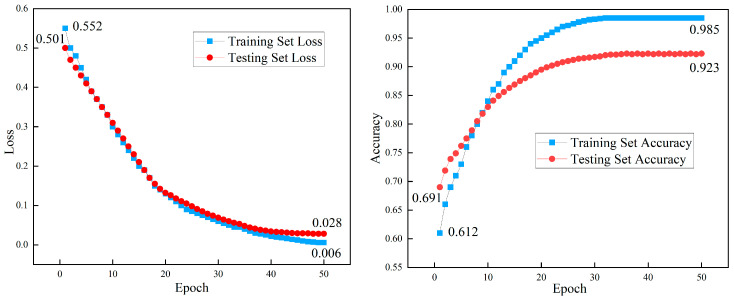
Accuracy graph of the training set and testing set over epochs.

Based on the constructed dataset and model, the corresponding changes in accuracy and loss of the training set and testing set with training epochs are shown in Figure 23.

The results show that the initial pre-trained model has an accuracy rate of over 60% for various category recognition, demonstrating the effectiveness of the initial parameters of the pre-trained model. Moreover, the model converges after approximately 30 epochs, with a relatively fast convergence speed, verifying the effectiveness of the model’s transfer training using the pre-trained model. Meanwhile, the training loss of the model gradually decreases with each iteration, and the error converges gradually. The loss of the training set eventually converges to 0.006, and the accuracy rate of the final test set reaches 92.3%.

To justify the selection of ResNet-18, comparative experiments were conducted using several representative models, including VGG-16, MobileNet-V2, and a traditional Support Vector Machine (SVM) classifier. All deep learning models were pre-trained on ImageNet and fine-tuned using the same training configuration (learning rate: 0.001, batch size: 32, epochs: 50). For SVM, features were extracted using a histogram of oriented gradients (HOG). The comparison results are presented in Table 1: Performance Comparison of Different Classification Models.

As shown in Table 1, ResNet-18 achieved the highest accuracy of 92.3% and F1-score of 0.912 among all tested models. Although VGG-16 has significantly more parameters, its performance (89.1%) was inferior to ResNet-18, likely due to overfitting on the relatively small dataset. MobileNet-V2, despite its lightweight architecture, achieved 87.5% accuracy, demonstrating reasonable performance but falling short of ResNet-18. The traditional SVM classifier yielded the lowest accuracy of 76.8%, indicating that deep learning approaches are more effective for capturing complex texture features in surrounding rock images. These results confirm that ResNet-18 provides the optimal balance between model complexity and classification performance for this application.

**Figure 24:**
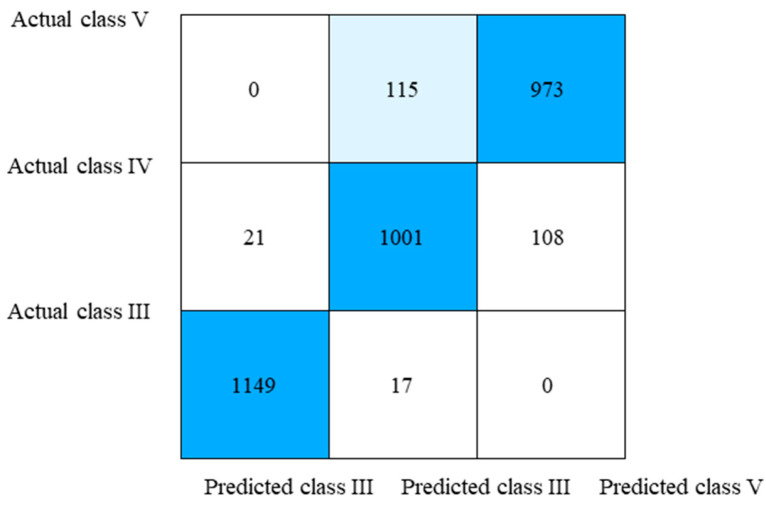
Confusion matrix of test results.

The confusion matrix of the surrounding rock classification results is shown in Figure 24. The confusion matrix analysis reveals the classification performance for each rock class. For class III, 98.5% of samples were correctly classified (recall), with only a small portion misclassified as class IV. For class IV, the recall rate is 88.6%, with misclassifications mainly occurring toward class V. For class V, the recall rate is 89.5%, with misclassifications only occurring toward class IV. The overall accuracy of 92.3% represents the ratio of all correctly classified samples to the total number of test samples, which differs from the per-class recall rates due to varying class sizes in the test set. The recognition accuracy for each category is basically stable, in line with the expected results.

To provide a more comprehensive evaluation of the classification performance, precision, recall, and F1-score were calculated for each surrounding rock class based on the confusion matrix. The results are presented in Table 2.

As shown in Table 2, class III surrounding rock achieved the highest F1-score of 0.977, indicating excellent classification performance with minimal confusion with other classes. Class IV and class V showed slightly lower but acceptable F1-scores of 0.888 and 0.901, respectively. The confusion between these two classes is attributed to their transitional characteristics in terms of joint development and weak layer distribution. The weighted average F1-score of 0.921 demonstrates the overall effectiveness of the proposed classification method.

**Figure 25:**
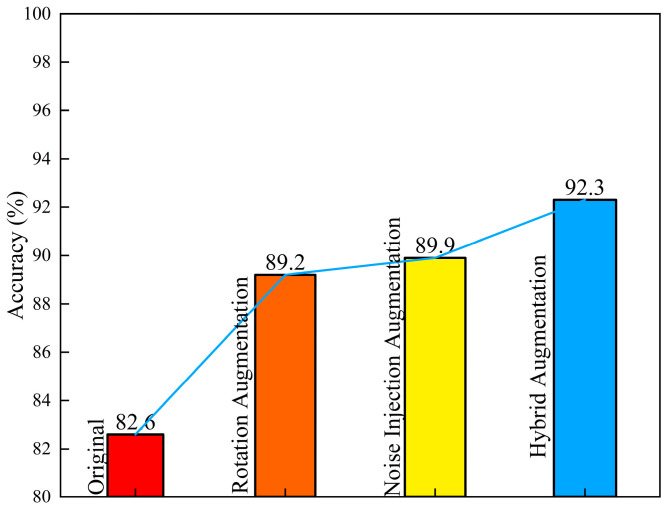
Accuracy of datasets with three augmentation methods.

This study compared the effects of four different image dataset enhancement methods on the training recognition results using the complete dataset. Each augmentation strategy was applied to all original images, and the resulting datasets were evaluated using the same 7:3 stratified train–test split and identical training configurations to ensure fair comparison. The results are shown in Figure 25. The accuracy of the original dataset was 82.6%. After rotation enhancement, it increased to 89.2%, and with noise enhancement, it further reached 89.9%. However, the mixed enhancement method significantly improved the accuracy to 92.3%. This indicates that for rock image samples, mixed enhancement can better enrich the data samples and improve the training accuracy of the model.

This study conducts research on the surrounding rock identification technology based on deep ResNet and transfer learning. It fully leverages the feature extraction advantages of deep ResNet and achieves rapid adaptation, efficient training and accurate identification of tunnel rock images through the transfer learning.

## 6. Conclusions

This study focuses on feature extraction and classification evaluation of tunnel surrounding rock in the Central Yunnan Water Diversion Project through image processing and analysis. Fractal theory is introduced to effectively evaluate the complexity of the surrounding rock and achieve accurate classification, along with a proposed method for calculating the modified surrounding rock strength. Furthermore, transfer learning is applied to the recognition of tunnel surrounding rock images, and an efficient identification model has been constructed successfully. The main conclusions are as follows:

(1) Based on image processing and fractal theory, features such as weak layer and joints in the surrounding rock can be effectively extracted and quantitatively characterized. A fractal dimension evaluation index is proposed to facilitate the standardized classification of different surrounding rocks. For the processed images, the distribution range of the fractal dimension evaluation index is 1.684–1.955 for class III surrounding rock, 2.192–2.443 for class IV, and 2.507–2.745 for class V, demonstrating clear classification boundaries and effective discriminative performance.
(2) By correcting the rebound hammer strength data with the extracted parameters from surrounding rock images, more accurate strength characteristics of the surrounding rock were obtained. Before correction, the strength value ranges were: 26.4–36.1 MPa for class III surrounding rocks, 18.5–31.7 MPa for class IV, and 10.3–21.8 MPa for class V, exhibiting overlapping ranges between different rock classes. After correction, the ranges became: 25.2–35.7 MPa for class III, 14.9–24.6 MPa for class IV, and 5.1–14.7 MPa for class V, demonstrating clear differentiation in strength characteristics among the various surrounding rocks. This strength correction method effectively captures the distinct strength characteristics of surrounding rocks belonging to different classes.
(3) The ResNet-18 model, enhanced with transfer learning, was applied to recognize surrounding rock images. Utilizing pre-trained weights from ImageNet effectively improved processing efficiency and feature extraction capability. It reduced training time and mitigated overfitting risks, thereby enhancing the model’s generalization performance. The surrounding rock recognition is fast and applicable. Comparative experiments demonstrated that a mixed data augmentation approach yielded the best performance, achieving an overall recognition accuracy of 92.3%.

Despite the promising results, this study has several limitations that should be acknowledged. First, the dataset was collected from a single tunnel project (Central Yunnan Water Diversion Project) with primarily granite and limestone geology. The geological conditions in other tunnel projects may differ significantly in terms of rock types, weathering degrees, and structural characteristics. Second, the current study focused on classes III, IV, and V surrounding rock, while classes I and II were not included due to limited sample availability at the study site. Third, the accuracy of the image-based strength correction method depends on the precision of weak layer segmentation. While Otsu’s thresholding method provides consistent results, minor segmentation inaccuracies may affect the calculated area proportions. However, the significant improvement in class separation (97.7% overlap reduction) suggests the achieved accuracy is sufficient for practical classification purposes.

However, the proposed framework demonstrates strong potential for generalization due to the following reasons: (1) The fractal theory and box-counting method are universal mathematical tools applicable to any rock surface image regardless of geological setting. (2) The image-based strength correction method relies on fundamental principles of area-weighted averaging, which is independent of specific rock types. (3) The transfer learning approach using pre-trained ResNet-18 has proven effective for various image classification tasks and can adapt to new geological conditions with minimal fine-tuning.

Future work will focus on: (1) validating the proposed framework on multiple tunnel projects with diverse geological conditions; (2) extending the classification system to include classes I and II surrounding rock; (3) developing a real-time field application system for on-site surrounding rock evaluation; (4) exploring multimodal fusion methods to integrate fractal features directly into the deep learning model for joint decision-making, potentially improving classification accuracy and robustness.

## Figures and Tables

**Table 1:** Performance comparison of different classification models.

Model	Parameters	Accuracy (%)	F1 Score	Training Time (min)
SVM (HOG)	-	76.8	0.752	5
MobileNet-V2	3.4M	87.5	0.861	35
VGG-16	138M	89.1	0.878	120
ResNet-18	11.7M	92.3	0.912	45

**Table 2:** Classification performance metrics for each rock class.

Class	Precision	Recall	F1-Score
III	0.970	0.985	0.977
IV	0.891	0.886	0.888
V	0.908	0.895	0.901
Weighted Average	0.920	0.923	0.921

## Data Availability

The raw data supporting the conclusions of this article will be made available by the authors on request.

## References

[1] Wang Y., Jing H., Su H., Xie J. (2017). Effect of a Fault Fracture Zone on the Stability of Tunnel-Surrounding Rock. Int. J. Geomech..

[2] Zhu W., Li S., Li S., Chen W., Lee C. (2003). Systematic Numerical Simulation of Rock Tunnel Stability Considering Different Rock Conditions and Construction Effects. Tunn. Undergr. Sp. Technol..

[3] Sun Z., Zhang D., Fang Q., Dui G., Tai Q., Sun F. (2021). Analysis of the Interaction between Tunnel Support and Surrounding Rock Considering Pre-Reinforcement. Tunn. Undergr. Sp. Technol..

[4] Liu B., Lin H., Chen Y. (2022). Deformation Characteristics of Bolted Rock Joints under Compression-Shear Load. Appl. Sci..

[5] Dorafshan S., Thomas J., Maguire M. (2018). Comparison of Deep Convolutional Neural Networks and Edge Detectors for Image-Based Crack Detection in Concrete. Constr. Build. Mater..

[6] Ren Y., Huang J., Hong Z., Lu W., Yin J., Zou L., Shen X. (2020). Image-Based Concrete Crack Detection in Tunnels Using Deep Fully Convolutional Networks. Constr. Build. Mater..

[7] Kang D., Benipal S., Gopal D., Cha Y. (2020). Hybrid Pixel-Level Concrete Crack Segmentation and Quantification across Complex Backgrounds using Deep Learning. Automat. Constr..

[8] Amir R., Radhakrishna A., Michele G., Katrin B. (2020). Comparison of Crack Segmentation Using Digital Image Correlation Measurements and Deep Learning. Constr. Build. Mater..

[9] Liu Z., Hai L. (2010). Experimental Investigation on the Deformation and Crack Behavior of Rock Specimen with a Hole Undergoing Uniaxial Compressive Load. Eng. Mech..

[10] Zhang G., Wu S., Guo P., Zhang S. (2023). Mechanical Deformation, Acoustic Emission Characteristics, and Microcrack Development in Porous Sandstone During the Brittle–Ductile Transition. Rock Mech. Rock Eng..

[11] Zhao S., Wang M., Yi W., Yang D., Tong J. (2022). Intelligent Classification of Surrounding Rock of Tunnel Based on 10 Machine Learning Algorithms. Appl. Sci..

[12] Lopes R., Betrouni N. (2009). Fractal and Multifractal Analysis: A Review. Med. Image Anal..

[13] Huang S., Yu S., Ye Y., Ye Z., Cheng A. (2022). Pore Structure Change and Physico-mechanical Properties Deterioration of Sandstone Suffering Freeze Thaw Actions. Constr. Build. Mater..

[14] Miao H., Yang X., Yin D., Zheng W., Zhang H., Zhang S., Liu Z. (2022). A Novel Defrosting Control Strategy with Image Processing Technique and Fractal Theory. Int. J. Refrig..

[15] Zhang X., Lin H., Liu H., Li S., Li J., Chen J. (2023). A Fatigue Constitutive Model for Rock Masses Based on Cross-Applications of Rheological Theory. Eur. J. Environ. Civ. Eng..

[16] Pang Y., Lin H., Cao P., Meng G. (2025). The Influence of Overlying High-Speed Rail Dynamic Loads on the Stability of Shield Tunnel Faces During Excavation. Appl. Sci..

[17] Aladejare A.E. (2020). Evaluation of Empirical Estimation of Uniaxial Compressive Strength of Rock Using Measurements from Index and Physical Tests. J. Rock Mech. Geotech..

[18] Zhang G., Zhang S., Guo P., Wu S. (2023). Acoustic Emissions and Seismic Tomography of Sandstone Under Uniaxial Compression: Implications for the Progressive Failure in Pillars. Rock Mech. Rock Eng..

[19] Chen C., Li O., Barnett A., Su J., Rudin C. (2019). This Looks Like That: Deep Learning for Interpretable Image Recognition. arXiv.

[20] Wang J., Cao A., Wu Z., Liu X., Li Z., Lin L., Liu X., Li H., Sun Y. (2023). Improved Surrounding Rock Classification Method for the Middle Rock Pillar of a Small Clear-Distance Tunnel. Appl. Sci..

[21] Kovalchuk A.V., Lebedev A.A., Shemagina O.V., Nuidel I.V., Yakhno V.G., Stasenko S.V. (2025). Enhancing Cascade Object Detection Accuracy Using Correctors Based on High-Dimensional Feature Separation. Technologies.

[22] Xie S., Lin H., Chen Y., Yao R., Sun Z., Zhou X. (2025). Hybrid Machine Learning Models to Predict the Shear Strength of Discontinuities with Different Joint Wall Compressive Strength. Nondestr. Test. Eval..

[23] Wang X. (2016). Deep Learning in Object Recognition, Detection, and Segmentation. Found. Trends. Signal..

[24] Xu Z., Ma W., Lin P., Shi H., Pan D., Liu T. (2021). Deep Learning of Rock Images for Intelligent Lithology Identification. Comput. Geosci..

[25] Wang H., Jiang W., Yang J., Xu Z., Zhi B. (2025). Network Intrusion Detection Integrating Feature Dimensionality Reduction and Transfer Learning. Technologies.

[26] Shao S., Song R., Wu Y., Zhang Z., Fu H., Peng Y., Li Z., Liu Y. (2025). Research on Intelligent Predictions of Surrounding Rock Ahead of the Tunnel Face Based on Neural Network and Longitudinal Deformation Curve. Appl. Sci..

[27] Zhu W., Braun B., Chiang L.H., Jose A. (2021). Investigation of Transfer Learning for Image Classification and Impact on Training Sample Size. Chemometr. Intell. Lab..

[28] Zhang W., Zhang W., Zhang G., Huang J., Li M., Wang X., Ye F., Guan X. (2024). Hard-rock Tunnel Lithology Identification Using Multi-scale Dilated Convolutional Attention Network Based on Tunnel Face Images. Front. Struct. Civ. Eng..

[29] Liu L., Song Z., Zhou P., Zhang J., Liu G. (2024). AI-based Rock Strength Assessment from Tunnel Face Images Using Hybrid Neural Networks. Sci. Rep..

[30] Fallahy S., Rezazadeh N. (2025). MARBLE-DA: Masonry Analysis with Robust, Batch-normalised, Label-free, Explainable Domain Adaptation for Crack Detection. J. Build. Eng..

[31] (2015). Standard for Engineering Classification of Rock Mass.

